# Left Atrial Thrombus in the Setting of Mitral Stenosis

**DOI:** 10.14797/mdcvj.1272

**Published:** 2023-08-24

**Authors:** Ola Abdelkarim, Yehia Saleh, Faisal Nabi

**Affiliations:** 1 Houston Methodist DeBakey Heart & Vascular Center, Houston Methodist Hospital, Houston, Texas, US

**Keywords:** mitral stenosis, left atrial thrombus, left atrial appendage thrombus

## Abstract

A 56-year-old man with no significant past medical history presented with exertional shortness of breath. Echocardiogram, cardiac magnetic resonance, and computed tomography showed mitral stenosis and a left atrial thrombus. Left atrial thrombus formation is a well-known complication of severe mitral stenosis that can lead to systemic thromboembolism. The patient underwent mitral valve replacement, left atrial thrombus resection, and left atrial appendage closure that resulted in significant improvement in breathing.

A 56-year-old man with no significant past medical history presented with exertional shortness of breath. On examination, vital signs were stable, and a mid-diastolic rumbling murmur was appreciated on auscultation. Electrocardiogram showed normal sinus rhythm with P mitrale. An echocardiogram (echo) showed a preserved ejection fraction, severe mitral stenosis, and a dilated left atrium with a large thrombus (5 × 3.8 cm). Cardiac magnetic resonance confirmed the echo findings with features consistent with a thrombus. Preoperative cardiac computed tomography showed that the thrombus originated from the left atrial appendage (Figure 1, Video 1). The patient underwent successful mitral valve replacement, left atrial thrombus resection, and left atrial appendage closure. Postoperatively, the patient’s shortness of breath significantly improved, and the follow-up echo showed a well-functioning mitral valve prosthesis.

**Figure 1:**
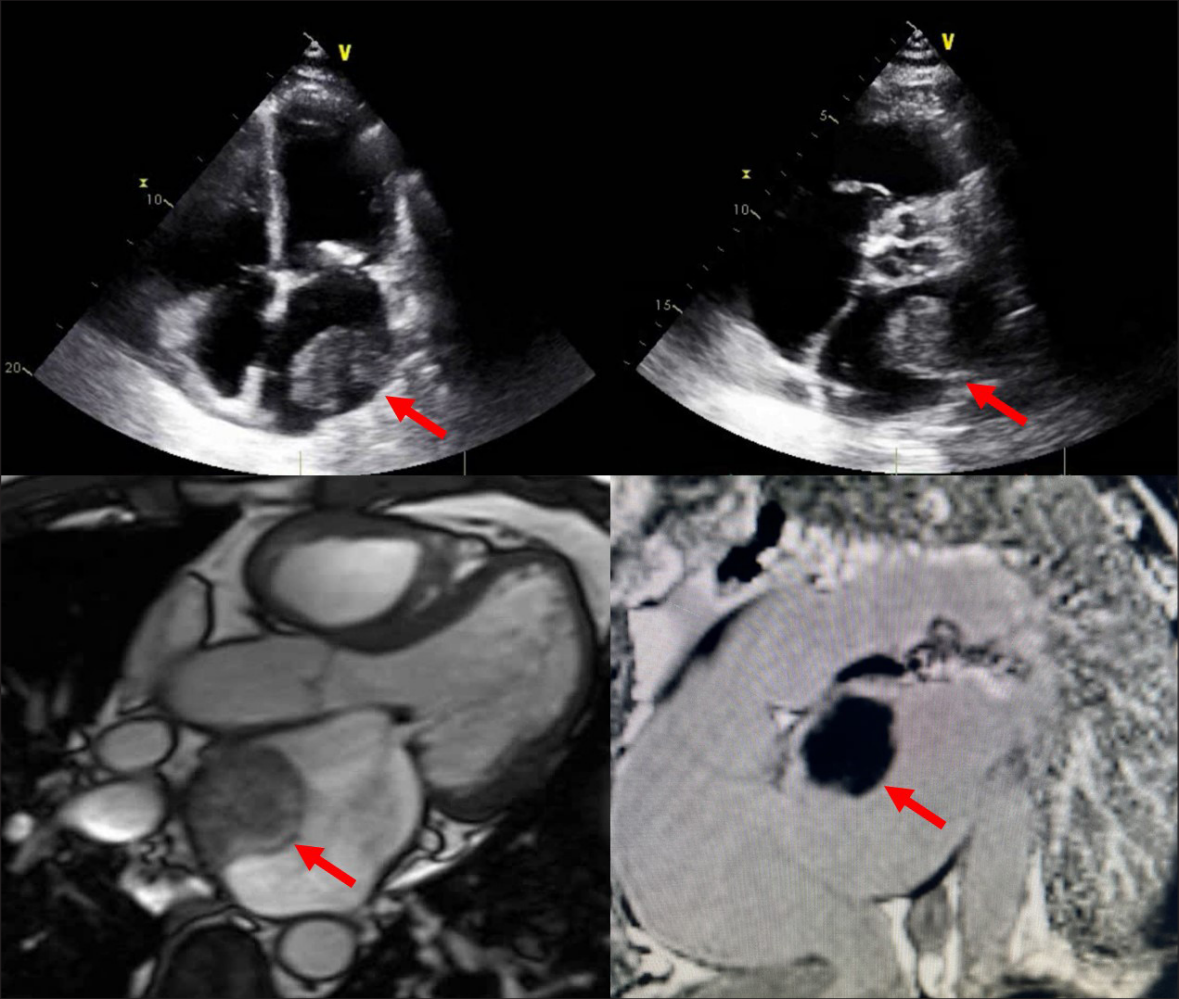
Echocardiography and magnetic resonance imaging showing a thrombus in the left atrial appendage.

**Video 1:** Echocardiogram, cardiac magnetic resonance, and computed tomography show mitral stenosis and a left atrial thrombus; see also at https://youtu.be/lG5d5c3nBwg.

Left atrial thrombus formation is a well-known complication of severe mitral stenosis that can lead to systemic thromboembolism. It occurs secondary to the significant blood stasis in the dilated left atrium. To decrease the risk of thromboembolic complications, anticoagulation is recommended in patients with mitral stenosis complicated by atrial fibrillation. Moreover, in patients with significant mitral stenosis in sinus rhythm, long-term anticoagulation should be considered when the echo shows dense spontaneous echocardiographic contrast or an enlarged left atrium. A recent multicenter randomized study showed that vitamin K antagonist (VKA) therapy led to lower cardiovascular events compared with rivaroxaban therapy in patients with rheumatic heart disease-associated atrial fibrillation. Hence, VKA therapy is the recommended anticoagulation strategy in this population.^1, 2, 3^
